# The Sensitivity, Specificity and Accuracy of Warning Signs in Predicting Severe Dengue, the Severe Dengue Prevalence and Its Associated Factors

**DOI:** 10.3390/ijerph15092018

**Published:** 2018-09-15

**Authors:** Mohd Hanief Ahmad, Mohd Ismail Ibrahim, Zeehaida Mohamed, Nabilah Ismail, Muhammad Amiruddin Abdullah, Rafidah Hanim Shueb, Mohd Nazri Shafei

**Affiliations:** 1 Department of Community Medicine, School of Medical Sciences, Universiti Sains Malaysia, Kubang Kerian, Kota Bharu 16150, Kelantan, Malaysia; haniefahmad@gmail.com (M.H.A.); drnazri@usm.my (M.N.S.); 2 Department of Microbiology & Parasitology, School of Medical Sciences, Hospital Universiti Sains Malaysia, Kubang Kerian, Kota Bharu 16150, Kelantan, Malaysia; zeehaida@usm.my (Z.M.); drnabilah@usm.my (N.I.); amiruddin@usm.my (M.A.A.); hanimkk@usm.my (R.H.S.)

**Keywords:** severe dengue, accuracy, warning signs, prediction, Malaysia

## Abstract

*Objectives:* To study Malaysian dengue clinical practice guideline (CPG) warning signs (WS) in predicting severe dengue (SD) and its associated factors among confirmed cases presented to a teaching hospital in north-eastern Malaysia in 2014. *Methods:* A cross-sectional study was performed in February 2015 using secondary data acquired from the hospital records. There were 2607 confirmed dengue cases presented to Hospital Universiti Sains Malaysia (HUSM) in 2014. Seven hundred patients were selected after proportionate stratified random sampling conducted according to the number of cases in 12 different months in 2014. Data were collected and analysed using SPSS version 22.0. *Results:* Severe dengue outcomes represented 4.9% of cases. The prevalence of any of WS in SD was 91.2%. The most common WSs prior to SD were persistent vomiting (55.9%), and abdominal pain/tenderness (52.9%). The most sensitive warning sign in detecting SD was abdominal pain (59%). Specificity of individual WS were generally good, especially of clinical fluid accumulation (99%), hepatomegaly (98%) and mucosal bleeding (93%). Factors associated with SD were persistent vomiting (Adjusted odds ratio (aOR)): 2.41), mucosal bleeding (aOR: 4.73) and haematocrit rise with rapid platelet drop (aOR: 2.74). *Conclusion:* A focus on sensitivity, specificity, predictive values and association of a number of particular WS should be emphasized in order to better predict severe dengue outcomes.

## 1. Introduction

Dengue has become a significant public health interest all over the world since the 1950s [1]. World Health Organization (WHO) predicts that 2.5 billion people were exposed to dengue and its complications. A current model calculates 390 million cases per year, with 96 million cases manifesting with at least some clinical presentations [2]. Almost 2.5% died from the infection [3]. Dengue affects tropical and subtropical nations around the world, especially in city and non-rural areas. In Malaysia, the reported dengue cases have generally been increasing in the recent years. In 2012, a total of 21,900 cases and 35 deaths were reported [4]. This was equivalent to approximately 76 cases per 100,000 people.

The 2009 WHO criteria categorized dengue corresponding to degrees of severity: dengue without warning signs; dengue with warning signs (abdominal pain, persistent vomiting, fluid accumulation, mucosal bleeding, lethargy, liver enlargement, increasing haematocrit with decreasing platelets); and severe dengue (dengue with severe plasma leakage, severe bleeding, or organ failure) [5]. Patients who recuperated directly after defervescence were believed to acquire non-severe dengue, but those whose situation worsened were more likely to develop WSs. These patients tended to improve with symptomatic treatments and fluids. However, further decline was labelled as severe dengue, though improvement was feasible if right and prompt treatment was performed.

The 2009 WHO guidelines recommended utilising warning signs (WS) as a gauge for disease course [3]. WHO advises patients with any WS be admitted to a hospital for observation and treatment. Malaysian Clinical Practise Guideline (MCPG) for Dengue Management in Adults has adopted these WS into the criteria for hospital admission or referral [6]. It is crucial to assess these WS capacity in predicting who will potentially progress to DHF/DSS (Dengue Hemorrhagic Fever/Dengue Shock Syndrome) in the most reliable, economical and fastest way, so as to prioritise limited resources.

Sensitivity of a WS in predicting severe dengue gauged the percentage of genuine cases of severe dengue that were rightly recognized as such. Specificity of a WS quantified the percentage of real non-severe dengue cases that were rightly spotted as such. Across different literatures, there were mixed results of sensitivity (Sn), specificity (Sp), positive predictive value (PPV) and negative predictive value (NPV) of the WHO 2009 WS in predicting severe dengue. One of the studies concluded that no WS was highly sensitive (>65%) in predicting SD but their specificity for SD was quite high (90%) with persistent vomiting, hepatomegaly, haematocrit rise and rapid platelet drop, clinical fluid accumulation, and any 3 or 4 WS [7].

Narvaez et al. (2011) however deducted that the revised WHO classification for severe dengue appears to have higher sensitivity and specificity to identify cases in need of heightened care [8]. Whereas Cavalcanti et al. (2014) reported lower sensitivity (60.4%) and lower specificity (35.5%) to capturing SD cases, with low PPV (61.5%) and low NPV (34.4%) [9].

There are a myriad of publications which have shown significant factors associated with severe dengue. Jayaratne et al. (2012) reported that the presence of 5 or more warning signs appears to be a predictor of SD [10]. Lymphocyte counts < 1500 cells/mm^3^, platelet counts < 20,000/mm^3^ and raised aspartate transaminase (AST) levels were associated with SD, and could be used to help identify patients who were likely to develop SD. Leo et al. (2011) concluded that persistent vomiting and ≥20% haematocrit change concurrent with platelet count < 20 × 10^9^/L were significantly associated with dengue death [11]. Whereas Thein et al. (2013) observed that the absence of myalgia and leukocytosis on admission were independently associated with fatality in their matched case-control study. They also found that fatalities were also commonly associated with co-morbidities [7].

A significant association was observed between the severity of thrombocytopenia and the age groups. Thrombocytopenia was found to be more severe in age groups of 6–10 years than in the older age group, and this difference was significant [12]. Jayashree et al. (2011) also pointed out that a significant association was observed between the severity of thrombocytopenia and the clinical presentation of SD. These findings were also seen in a few other studies [13,14,15]. Kularatne et al. (2009) also found that the day of the illness and patient age as the independent predictors of platelet count change [16].

This study is paramount because it answers questions regarding the prevalence of SD in a tertiary hospital, and the predictive value of the adopted WS in predicting SD in Malaysia. It also looks into factors associated with SD in Malaysia. There were already 166 articles, published between the years 2000–2013 and pertaining to dengue in Malaysia, discovered from a search through a collection of data [17]. However, out of these papers, only a few of them had looked into the capacity of using these WS to guide hospital admission and predict disease progression. Moreover, almost none of them have ever gone into detail in studying the validity and predictive values of these WS, especially in forecasting severe dengue cases in north-eastern Malaysia. What made this study very noticeable compared to others was its focus on the WS in predicting severe dengue in the north-eastern region of Peninsular Malaysia, which received heavier rain during November-to-February monsoon season. Temperature, rainfall and humidity were shown to affect the prevalence of dengue and its trend [18]. The severity of dengue infections was also influenced by dengue serotypes [19]. North-eastern region of Peninsular Malaysia showed a different distribution of dengue serotype compared to the West-Coast region and East Malaysia region [20,21,22,23]. This study would further shed light on the probable impact these geographical-based differences would bring to the prevalence of severe dengue, as well as on its WS and symptoms.

## 2. Materials and Methods

### 2.1. Subjects

The study was carried out in a teaching hospital in north-eastern Malaysia. It has become a tertiary reference centre to other hospitals in north-eastern Malaysia. The records of all lab-confirmed patients, either by NS1 or dengue serology, presented to the hospital in 2014 were the sampling frame. Those who were diagnosed with co-infection like leptospirosis or referred to another centre or hospital were excluded from the study. There were 2607 confirmed dengue cases presented to the hospital in 2014. Seven hundred patients were randomly selected, proportionate to the number of cases in twelve different months in 2014.

### 2.2. Measurements

This study involved secondary data collection. The permission to use related data was obtained from the administrative office following the approval from the Universiti Sains Malaysia (USM) Human Ethics Committee. Patients’ records were then scrutinized for data required.

### 2.3. Ethical Approval

This study was approved by the National Medical Research Registry (NMRR-14-1791-23655) and Ethics Committee and Human Research Ethics Committee Universiti Sains Malaysia (USM/JEPeM/14120519). The confidentiality of the data had been strictly maintained. Only the author and supervisors had the access to the data available. Later, the reporting and publications were carried out with no respondents’ name mentioned.

### 2.4. Data Analysis

Data were entered and analysed using the Statistical Package for Social Sciences (version 22, IBM SPSS Company, Armonk, NY, USA). Descriptive statistics were computed for all variables. Sensitivity (Sn), specificity (Sp), positive predictive value (PPV) and negative predictive value were measured using the formula as shown in Table 1.

Logistic regression analysis was conducted to identify the significant associated factors with SD. The fitness of the final multiple logistic regression model was further tested using the Hosmer-Lemeshow test, the classification table and the area under Receiver Operating Characteristic (ROC) curve. A fit model would demonstrate *p*-value of more than 0.05 for Hosmer-Lemeshow test, overall correctly classified percentage of more than 80% in the classification table, and an area under the ROC curve of more than 70%. The associations of independent variables with severe dengue were expressed as odds ratios (ORs) with 95% confident intervals (CI). A statistical probability level of *p* < 0.05 was considered significant.

## 3. Results

There were 700 patients recruited in the present study. The median (IqR) age of the patients was 25 (23) years old (the detail of socio-demographic of respondents refer to Table 2). In term of the outcomes (Table 3), 34 (4.9%) patients acquired severe dengue (SD) with 95% CI of 3 to 6%. Among those 34 severe dengue cases, 85.3% had severe plasma leakage, 11.8% had severe bleeding and 23.5% had severe organ involvement.

Table 4 demonstrated the existence of warning signs at presentation. Among the 700 patients, 47.3% of the patients had any of seven warning signs at presentation. A higher proportion of any WS at presentation (52.3%) was seen among 304 patients who had been admitted. And the percentage of any WS at presentation was the highest among 34 patients who developed SD (91.2%). The three most prevalent warning signs that occur prior to severe dengue cases were vomiting (55.9%), abdominal pain (52.9%), and lethargy (35.3%).

The capacities of warning signs to anticipate SD in 700 patients were illustrated in Table 5. The sensitivity to detect SD was 91.2% by having any of the seven warning signs. The sensitivity of the warning signs sensibly reduced with the increasing number of warning sign combinations (6% sensitivity for 5 warning signs combination). The most sensitive warning signs in detecting SD were abdominal pain (59%) and vomiting (56%). In term of specificity, clinical fluid accumulation, hepatomegaly and mucosal bleeding were the individual warning signs with the highest specificity (99%, 98% and 93% respectively). More combinations of warning signs indicated higher specificity (99% specificity of 5 WSs combination compared to only 71% specificity of only one WS). Positive predictive value (PPV) of clinical fluid accumulation was the highest (58%) among the seven WS, compared to only 9% in vomiting, which had the lowest PPV. A combination of 5WSs can only reach 40% PPV, with the trend seemed not related to the number of WS combination. However, the negative predictive value (NPV) of the WS were consistently 95% or higher in all of the individual WS, and in all of the available combinations of WS.

Simple logistic regression was conducted to explore the factors associated with severe dengue. In our study, there were no socio-demographic factors (including age, sex and ethnicity) found to be statistically significant (<0.25) with severe dengue. As also shown in Table 6, there was no significant association between reported co-morbidities such as diabetes mellitus (*p* = 0.674) and hypertension (*p* = 0.868) with severe dengue. However, all of the WS were associated with severe dengue. Using this method of univariable analysis, most of the WS showed strong associations (*p* < 0.001).

As demonstrated in Table 7, the significant factors associated with severe dengue were persistent vomiting (aOR: 2.41; 95% CI: 1.16, 4.99; *p* = 0.018), mucosal bleeding (aOR: 4.73; 95% CI: 2.09, 10.69; *p* < 0.001) and HCT rise and rapid platelet drop (aOR: 2.74; 95% CI: 1.21, 6.19; *p* < 0.015), after controlling confounders in multiple logistic regression. The other four WS, which were significant during simple logistic regression, were found to be not significant. The Hosmer and Lemeshow test for fitness of the model was not significant with a *p*-value of 0.093, indicating that the model was fit. The model fitness was also advocated by classification table which indicated that 95.1% were correctly classified; and by area under Receiver Operating Characteristic (ROC) curve which was 0.742, implying acceptable discrimination of the model. All the assumptions in multiple logistic regression were met. There was no multicollinearity and interaction issue found.

## 4. Discussion

Of 700 cases in our study, 49.7% were male. Anker and Arima (2011) showed a consistent and significant excess among males based on reported dengue cases from national surveillance systems in 6 Asian countries including Malaysia [25]. The difference between their study and our study lay in the inclusion of paediatric patients in our study. Adult males were more mobile and more exposed to dengue risk. This effect was probably buffered by a paediatric population whose mobility was lesser than the adult, hence lesser exposure risk to dengue. The predominance of Malay in our study was explained by a large Malay population in northeastern Malaysia, which exceeded 90% proportion [26].

With a median age of 25 years old and a majority (75.4%) of patients in our study more than 15 years of age, the findings were consistent with a study done by Mohd-Zaki et al. (2014) which demonstrated that there has been a shift in the age range predominance from children to adults [27]. They had conducted a literature survey and analysis to describe the epidemiology of dengue disease in Malaysia between 2000 and 2012. They discovered there was a non-linear increase in the number of reported cases from 7103 in 2000 to 46,171 in 2010. However, the overall increase in dengue disease was accompanied by a rise in the number, but not the proportion, of severe cases.

In our study, 396 patients were managed wholly as outpatients, while the remaining 304 (43.4%) were admitted for varied time periods. In the other Asian countries, almost 14% of patients were seen in outpatients, while in Latin American nations, the number ranged between 20% and 50% [1]. The statistics relied on a number of factors, such as the number of available beds, quality of health service, health care accessibility in that area and health-seeking behaviour.

### 4.1. The Prevalence of Severe Dengue

Our results showed that the prevalence of SD was 4.9%, which was quite similar to the previous studies by Zakaria et al. (2014) and Zhang et al. (2014) with the prevalence rate of 4.6% and 5.6% respectively [28,29]. However, the homogeneity of severe dengue prevalence demonstrated in these studies was not consistent with other studies which showed higher prevalence rate. A study on traveller’s dengue in Germany showed a prevalence rate of 11%, whereby the dengue infection had been imported from various regions of endemicity, such as South East Asia, India and America [14].

In another previous study, the prevalence for SD was 16.5% [7]. However, the researchers in this study included only patients with a positive dengue polymerase chain reaction (PCR) during the early febrile viremic phase of their illness. So, the difference might have played an important role in influencing the prevalence rate, as this study did not proceed with follow-up confirmatory serology test.

### 4.2. The Prevalence of Warning Signs

In the present study, the three most common warning signs of severe dengue infection were vomiting, abdominal pain and lethargy. This result was similar to other studies [30,31]. However, in a study by Roy et al. (2013), hepatic manifestations were more prevalent in severe dengue infection in children age 2 months to 14 years [32]. In the study, they divided the study population into three categories, which were dengue without warning signs, dengue with warning signs and severe dengue. They had a more detailed investigation into hepatic manifestations of the patients and asked questions such as whether there were hepatomegaly, hepatic tenderness, splenomegaly, jaundice, hepatic encephalopathy or raised aspartate transaminase (AST) and alanine transaminase (ALT).

A study done in five adult public hospitals in Singapore revealed that the commonest warning sign at presentation (within 7 days of diagnosis) that led to dengue death was lethargy, which existed in 39.3% of all cases resulting in death [11]. Their study populations were only 27 fatal cases in 2004 to 2008. Although it was a small study, it demonstrated the high prevalence of lethargy presentation before the patients succumbed to death. Apart from lethargy, another of the most common warning signs prior to death were haematocrit change of ≥20% with concurrent platelet count of <50 × 10^9^/L. This factor was associated with the shortest interval to death, at a median of 3 days.

### 4.3. Validity and Predictive Value of Warning Signs

In term of the individual warning signs sensitivity, Thein et al. (2013) showed that their individual warning signs were less sensitive, with only abdominal pain and lethargy having more than 20% sensitivity [7]. In comparison, our study demonstrated that out of seven warning signs, only hepatomegaly had a sensitivity of less than 25%. This discrepancy in individual warning signs sensitivity can only be explained by further research to look into the effects of demographic difference towards clinical manifestation. The predominant race in the study population by Thein et al. (2013) was Chinese (73.7%), compared to the Malay majority in the present study.

It was also interesting to note that the sensitivity of a 4 WS combo was higher (15%) than a 3 WS combo (3%) in predicting SD. Generally, the sensitivity declines with more combinations of diagnostic criteria. This rather unique finding could be explained by small SD samples available for this study using prevalence study design. A bigger pool of SD samples may see an increment in the sensitivity of the 3 WS combo to be above 15%.

In a study by Leo et al. (2013), the two most sensitive warning signs in detecting severe dengue were mucosal bleed (64%) and abdominal pain (34%). The study enrolled 499 confirmed dengue cases, with the inclusion criteria of 18 years and above, and an exclusion criterion of pregnant women. The study also excluded lethargy from their warning signs criteria. Their findings regarding the sensitivity of individual warning signs were quite patchy, as shown by 0% sensitivity value of clinical fluid accumulation and hepatomegaly [33].

There were limited studies that discussed individual warning signs’ sensitivity, specificity, PPV and NPV in detecting severe dengue. Many studies discussed the sensitivity and specificity of WHO warning signs in general. For example, two previous studies used generalized linear models (GLM) with a logit link function and binomial errors techniques to produce sensitivity and specificity scores [34,35].

In addition, Thein et al. (2013) also made a similar observation in their study about the high specificity of hepatomegaly (>90%) and clinical fluid accumulation. Poor PPV of individual WS in our study was also seen in their study, with only slight differences, whereby the mean PPV of 7 WS was 22.7% in our study, compared to 12.1% in theirs [7]. However, the most prominent finding that was homogenously shared between our studies was the high NPV value (>85%) of most individual WS and varying number of their combination.

### 4.4. Factors Associated with Severe Dengue

Our study illustrated that there was no association between sex and severe dengue. In contrast, Anders et al. (2011) found that females had a higher risk for DSS [36]. The discrepancy was possibly due to different socio-demographic characteristics and methodology used. They studied 132,480 dengue patients admitted to three referral hospitals in Vietnam, whereby our study included all hospitalized patients and outpatients. Males were more likely to be exposed to dengue as they were more mobile and socially ambulatory. Due to the same mobility reason, they had better access to hospitals and earlier treatment. However, females who were hospitalized might already indicate severe illnesses which were more likely to progress to severe dengue due to late treatment.

Having diabetes mellitus did not increase the risk of contracting severe dengue according to our study. This finding was in agreement with Mahmood et al. (2013) [37]. However, the finding was not consistent with the previous studies by Figueiredo et al. (2010) [38] and Pang et al. (2012) [39]. These two studies used case-control as their study design. However, those studies were prone to selection bias as their study population were only among hospitalized patients. This explanation was also the possible explanation for hypertension.

In our multivariable analysis, the factors significantly associated with severe dengue were persistent vomiting, mucosal bleeding and HCT rise with rapid platelet drop. This strength of association between patients who had persistent vomiting with severe dengue was also found in other studies [34,40]. Gupta et al. (2011) also showed a significant association between mucosal bleeding and severe dengue [41]. The associations between severe dengue with HCT rise with rapid platelet drop were also shown in another study [11].

Aung et al. (2013) showed that having a haematocrit of >2% above the reference range was associated with the severe clinical manifestation of adult dengue cases in Thailand [42]. Similar associations were also seen by other previous studies [43,44]. Phuong et al. (2004) found the median haematocrit value for DHF in Vietnamese children was 48% [43]. Meanwhile, Gomber et al. (2001) defined the haematocrit value diagnostic of DHF in Indian children at 36.3%, with sensitivity and specificity of 60% and 94% respectively [45]. In another study, it was also observed that a fluctuation of 20% in the haematocrit values has a highly significant association with DHF/DSS [44].

### 4.5. Limitations

There were a number of limitations in our study. Age of the respondent might become a bias to elicit the symptoms such as abdominal pain among infants. However, the abdominal pain/tenderness is important to be considered because it may depict an overall picture of dengue manifestation. Furthermore, the problem’s influence on the result of the analysis was more likely to be minimal, as the number in this age group was small (2.3%). However, having said that, this issue was still considered a limitation in this current study. As they could influence the presentations and severity of a dengue infection, secondary infection status and dengue serotype status were two of the important variables not accessible for our data analysis. Although unavailability of these kind of data did not actually affect the fitness of our regression model, the missing variable would definitely further improve the model and enlighten us on their effect in severe dengue developments in the North-Eastern region of Peninsular Malaysia, had the variables been accessible for analysis. Missing data was also one of the issues in a study using secondary data, although the extent of the problem was trivial in our study. To overcome this problem, one of our exclusion criteria in this study was the presence of missing data of more than 30%. Clinical skills of the medical doctors attending and treating dengue cases in HUSM were another factor which was not controlled in our study. As a tertiary centre cum teaching hospital, HUSM had a large number of fresh housemen and young doctors whose skills in identifying dengue clinical presentations or warning signs might not be as good as the registrars or the specialists. Although there were senior doctors who could double check these young doctors’ work, the information which had been written in the clinical folder of a patient might not have been corrected in most of the situations. This situation could lead to information bias during its transfer from the folder into the proforma.

## 5. Conclusions

The prevalence of severe dengue in our study was 4.9%. This prevalence rate was consistent with the other studies’ findings. The three most common warning signs in severe dengue cases were persistent vomiting, abdominal pain/tenderness and lethargy. Factors significantly associated with severe dengue in multivariable analysis were three: persistent vomiting, mucosal bleeding and HCT rise with rapid platelet drop. The findings were consistent with some other studies’ findings.

## Figures and Tables

**Table 1:** Formula for Sensitivity, Specificity, Positive Predictive Value and Negative Predictive Value.

Variables Used	SD	Non-SD	Total
Presence of WS	a	b	a + b
Absence of WS	c	d	c + d
Total	a + c	b + d	a + b + c + d
Example of the calculation			
Sensitivity = a/(a + c)			
Specificity = d/(b + d)			
PPV = a/(a + b)			
NPV = d/(c + d)			
Example: For any number of warning signs, a = 31, b = 300, c = 3, d = 366.			
For one warning sign only, a = 12, b = 155, c = 22, d = 511			
For two warning signs only, a = 11, b = 105, c = 23, d = 561			

**Table 2:** Socio-demographic characteristics of the respondents (*n* = 700).

Variable	*n* (%)
Age group	25 (23.00)
≤15 *	172 (24.6)
>15	528 (75.4)
Sex	
Men	348 (49.7)
Women	352 (50.3)
Ethnicity	
Malay	662 (94.6)
Others	38 (5.4)
Locality	
Kota Bharu	596 (83.7)
Bachok	62 (8.9)
Others	52 (7.4)

* based on a study by Malavige et al. (2004) [24].

**Table 3:** Clinical characteristics of the patients (*n* = 700).

Variable	*n* (%)
Place presented to	
HUSM Ward	304 (43.4)
Emergency & Trauma Unit	260 (37.2)
Clinics	136 (19.4)
Median days of fever at presentation (inter-quartile range)	4 (3.00)
Median days hospitalized (inter-quartile range)	3 (3.00)
Severe Dengue	34 (4.9)
Severe Plasma Leakage	29 (85.3)
Severe Bleeding	4 (11.8)
Severe Organ Involvement	8 (23.5)
Median platelet count at presentation (inter-quartile range)	152 (88.00)
Median HCT count at presentation (inter-quartile range)	38.9 (6.30)

**Table 4:** Prevalence of warning signs at presentation.

Warning Signs (WSs)	*n* (%)		
	Overall *n* = 700	All Inpatients *n* = 304	SD * n = 34
Individual WS			
Abdominal pain or tenderness	154 (22.0)	60 (19.7)	18 (52.9)
Persistent Vomiting	199 (28.4)	54 (17.8)	19 (55.9)
Hepatomegaly	10 (1.4)	19 (6.3)	2 (5.9)
Clinical fluid accumulation	8 (1.1)	15 (4.9)	5 (14.7)
Mucosal bleeding	44 (6.3)	23 (7.6)	8 (23.5)
Abnormal platelet with abnormal HCT	33 (4.7)	71 (23.4)	3 (8.8)
Lethargy	112 (16.0)	18 (5.9)	12 (35.3)
WS Count			
Any number of seven WSs	331 (47.3)	159 (52.3)	31 (91.2)
Any one WS only	167 (23.9)	95 (31.3)	12 (35.3)
Any two WSs combo only	116 (16.6)	39 (12.8)	11 (32.4)
Any three WSs combo only	34 (4.9)	17 (5.6)	1 (2.9)
Any four WSs combo only	11 (1.6)	4 (1.3)	5 (14.7)
Any five WSs combo only	3 (0.4)	4 (1.3)	2 (5.9)
Any six WSs combo only	0	0	0
All seven WSs combo only	0	0	0

* SD severe dengue.

**Table 5:** Performance of World Health Organization (WHO) Warning Signs (WS) in predicting SD.

Warning Signs (WS)	Sn	Sp	PPV	NPV
Individual WS				
Abdominal pain or tenderness	0.59	0.77	0.12	0.97
Persistent Vomiting	0.56	0.71	0.09	0.97
Hepatomegaly	0.24	0.98	0.35	0.96
Clinical fluid accumulation	0.32	0.99	0.58	0.97
Mucosal bleeding	0.32	0.93	0.20	0.96
Haematocrit rise and rapid platelet count drop	0.29	0.89	0.12	0.96
Lethargy	0.44	0.85	0.13	0.97
WS Count				
Any number of seven WSs	0.91	0.51	0.09	0.99
Any one WSs only	0.35	0.77	0.07	0.96
Any two WSs combo only	0.32	0.83	0.09	0.96
Any three WSs combo only	0.03	0.93	0.02	0.95
Any four WSs combo only	0.15	0.98	0.28	0.96
Any five WSs combo only	0.06	0.99	0.40	0.95

**Table 6:** Simple logistic regression analysis of factors associated with SD.

Warning Signs (WS)	Crude OR (95% CI)	Wald Stat	*p*-Value
Age	1.00 (0.98,1.02)	0.06	0.806
Sex (Female)	0.77 (0.39,1.54)	0.54	0.462
Ethnicity (non-Malay)	1.75 (0.51,5.99)	0.78	0.376
Reported Diabetes	1.37 (0.31,6.01)	0.18	0.674
Hypertension	0.88 (0.21,3.81)	0.03	0.868
Abdominal pain	4.87 (2.40,9.88)	19.29	<0.001
Persistent vomiting	3.15 (1.57,6.33)	10.40	0.001
Hepatomegaly	13.35 (5.20,34.30)	29.00	<0.001
Clinical Fluid Accumulation	39.34 (14.46,107.05)	51.68	<0.001
Mucosal bleeding	6.60 (3.03,14.39)	22.50	<0.001
Haematocrit rise and rapid platelet count drop	3.49 (1.60,7.60)	9.93	0.002
Lethargy	4.416 (2.17,8.98)	16.85	<0.001

**Table 7:** Multiple logistic regression analysis of factors associated with SD.

Variable	Crude OR ^a^	Adjusted OR ^b^	Wald Stat ^b^ (df)	*p*-Value ^b^
	(95% CI)	(95% CI)		
Persistent vomiting			5.63 (1)	0.018
No	1.00	1.00		
Yes	3.15 (1.57,6.33)	2.41 (1.16, 4.99)		
Mucosal bleeding			13.97 (1)	<0.001
No	1.00	1.00		
Yes	6.60 (3.03,14.39)	4.73 (2.09,10.69)		
Haematocrit rise and rapid platelet count drop			5.88 (1)	0.015
No	1.00	1.00		
Yes	3.49 (1.60,7.60)	2.74 (1.21,6.19)		

^a^ Simple logistic regression; ^b^ Forward LR and Backward LR Multiple Logistic Regression model was applied. Multicollinearity and interaction term were checked and not found. Hosmer-Lemeshow test (*p* = 0.093), classification table (overall correctly classified percentage = 95.1%) and area under ROC curve (74.2%) were applied to check the model fitness.
